# A continuous learning approach to brain tumor segmentation: integrating multi-scale spatial distillation and pseudo-labeling strategies

**DOI:** 10.3389/fonc.2023.1247603

**Published:** 2024-01-08

**Authors:** Ruipeng Li, Jianming Ye, Yueqi Huang, Wei Jin, Peng Xu, Lilin Guo

**Affiliations:** ^1^ Department of Urology, Hangzhou Third People’s Hospital, Hangzhou, China; ^2^ Department of Oncology, First Affiliated Hospital, Gannan Medical University, Ganzhou, China; ^3^ Department of Psychiatry, Hangzhou Seventh People’s Hospital, Hangzhou, China; ^4^ School of Medical Technology and Information Engineering, Zhejiang Chinese Medical University, Hangzhou, China; ^5^ Third Affiliated Hospital, Zhejiang Chinese Medical University, Hangzhou, China

**Keywords:** brain tumor segmentation, continuous learning, multi-scale spatial distillation, pseudo-labeling, feature extraction

## Abstract

**Introduction:**

This study presents a novel continuous learning framework tailored for brain tumour segmentation, addressing a critical step in both diagnosis and treatment planning. This framework addresses common challenges in brain tumour segmentation, such as computational complexity, limited generalisability, and the extensive need for manual annotation.

**Methods:**

Our approach uniquely combines multi-scale spatial distillation with pseudo-labelling strategies, exploiting the coordinated capabilities of the ResNet18 and DeepLabV3+ network architectures. This integration enhances feature extraction and efficiently manages model size, promoting accurate and fast segmentation. To mitigate the problem of catastrophic forgetting during model training, our methodology incorporates a multi-scale spatial distillation scheme. This scheme is essential for maintaining model diversity and preserving knowledge from previous training phases. In addition, a confidence-based pseudo-labelling technique is employed, allowing the model to self-improve based on its predictions and ensuring a balanced treatment of data categories.

**Results:**

The effectiveness of our framework has been evaluated on three publicly available datasets (BraTS2019, BraTS2020, BraTS2021) and one proprietary dataset (BraTS_FAHZU) using performance metrics such as Dice coefficient, sensitivity, specificity and Hausdorff95 distance. The results consistently show competitive performance against other state-of-the-art segmentation techniques, demonstrating improved accuracy and efficiency.

**Discussion:**

This advance has significant implications for the field of medical image segmentation. Our code is freely available at https://github.com/smallboy-code/A-brain-tumor-segmentation-frameworkusing-continual-learning.

## 1 Introduction

Brain tumors, characterized by abnormal growths in brain tissue, represent a significant medical challenge due to their impact on morbidity and mortality worldwide. They can manifest in various forms, ranging from benign to malignant, the latter being particularly aggressive and prone to metastasis (1). The complex etiology of brain tumors includes factors such as radiation exposure, genetic predisposition, and family history, emphasizing the need for early detection and accurate diagnosis (2).

In the field of brain tumor diagnostics, magnetic resonance imaging (MRI) has emerged as a superior modality to computed tomography (CT) due to its improved spatial resolution and soft tissue contrast. This makes MRI essential for preoperative assessment, therapeutic management, and survival prediction in brain tumor cases (3). However, the traditional approach of manual segmentation in MRI scans, while the gold standard, suffers from inherent inefficiencies and subjective variability, necessitating the exploration of automated techniques (4, 5).

In recent years, deep learning models, such as those proposed by Ma et al. (6), have achieved significant success in automatic brain tumor segmentation. These models excel at capturing both local and global contextual features, but often struggle with vanishing gradients and overfitting, especially in deeper network layers. Kumar et al. (7) addressed these issues by combining ResNet50 with global average pooling to enhance tumor classification for various tumor types.

**Figure 1:**
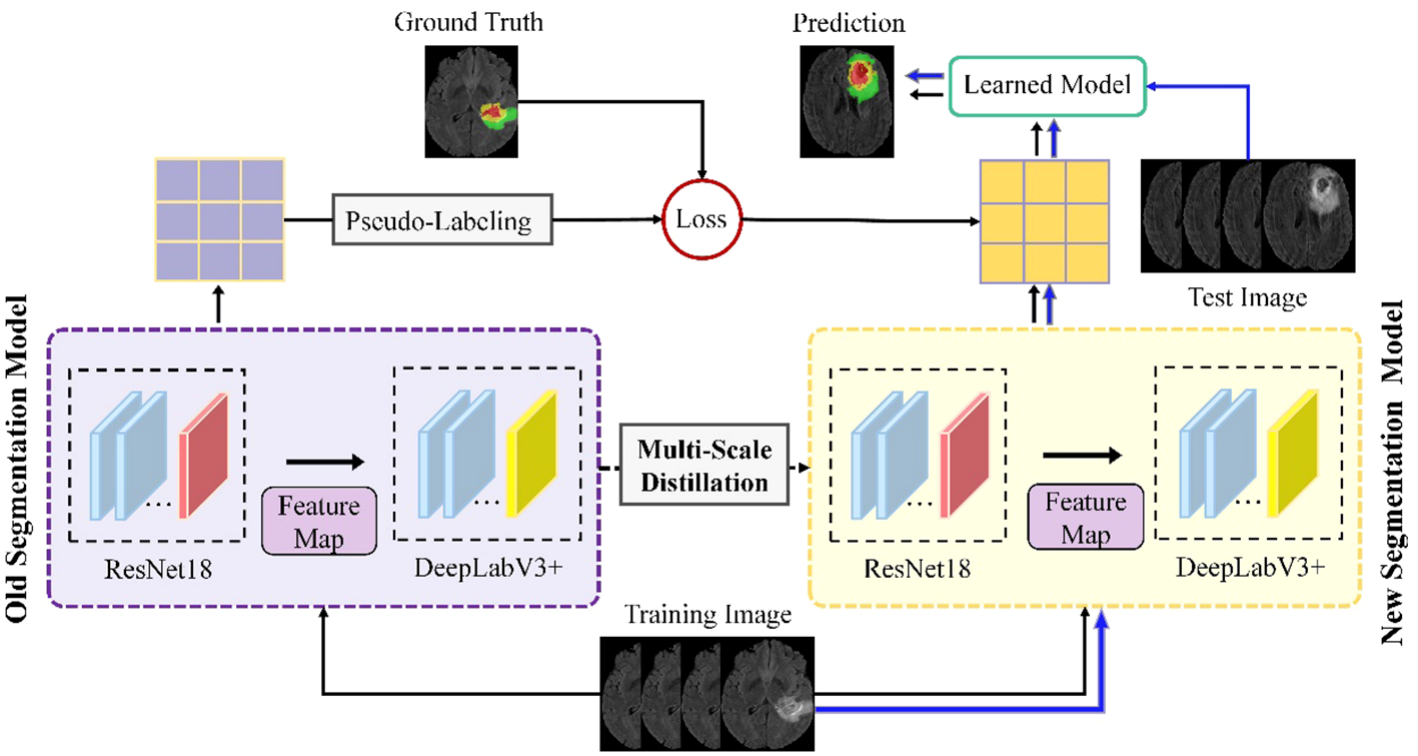
Schematic of the proposed continuous learning network for brain tumor segmentation.

Building on these foundations, our study introduces an advanced continuous learning framework for brain tumor segmentation from MRI images, as shown in **Figure 1**. Our methodology differs from existing techniques by integrating multi-scale spatial distillation and pseudo-labeling strategies. This approach not only overcomes the limitations of vanishing gradients and overfitting seen in previous models, but also addresses the issue of catastrophic forgetting - a common challenge in continuous learning models. Unlike traditional methods that rely on preserving subsets of training data or expanding the network architecture for new classes, our multiscale spatial distillation method focuses on preserving spatial relationships within the data. In addition, our confidence-based pseudo-labeling technique refines the segmentation process, particularly for non-tumor tissues, thereby improving the overall accuracy and reliability of the segmentation. We provide a comprehensive evaluation of our framework on several datasets, including BraTS2019, BraTS2020, BraTS2021, and a private dataset. This evaluation demonstrates the robustness of our model and its potential for clinical application, setting it apart from existing segmentation methods in terms of adaptability and performance.

## 2 Related works

### 2.1 Conventional brain tumor segmentation

In the early days of medical image segmentation, conventional machine learning algorithms were widely used because deep learning algorithms had not yet gained widespread popularity. Huang et al. (8) presented a semi-supervised learning algorithm using a unique image transformation strategy, combining a probabilistic deep neural network with an evidential neural network for dual evidence-based segmentation integrated by Dempster’s rule. Qin et al. (9) developed an unsupervised domain adaptation framework for brain tumor segmentation in MRI images, using dual student and adversarial training techniques to align feature representations and introducing a “cross-coordination constraint” to improve prediction confidence. Barzegar and Jamzad (10) proposed a semi-supervised unified framework for multi-label segmentation, which addresses the limitations and training requirements of atlas-based segmentation by reformulating the segmentation problem as a Markov Random Field energy optimization on a parametric graph, thereby improving accuracy and reducing computational burden. Bonte et al. (11) used a random forest model to represent different tumor tissues using only two MRI sequences, T1-CE and FLAIR MRI. They calculated local texture and abnormality maps and achieved good results in segmenting enhancing tissue, tumor core, and the whole abnormal region in high-grade gliomas. In another study, Kaur et al. (12) proposed a hybrid multilevel thresholding technique that combines intuitionistic fuzzy sets and Tsallis entropy to select tumor regions in MR images with blurred boundaries and poor contrast, further improving the segmentation speed and accuracy. These studies highlight the effectiveness of traditional machine learning techniques in medical image segmentation and lay the foundation for the development of more advanced deep learning-based methods.

### 2.2 Deep learning-based approaches

In recent years, advances in deep learning-based network models have contributed significantly to progress in the field of medical image segmentation. Bouchaour and Mazouzi (13) proposed a deep learning-based method for brain MRI tumor segmentation, utilizing an ensemble of CNNs to process segmented MRI volumes into sub-images for efficient voxel classification. This approach, focusing on local voxel patterns, greatly accelerates training and prediction, enhancing its suitability for real-time diagnostic applications. In a separate development, Zhang et al. (14) introduced the Hierarchical Multi-Scale Segmentation Network (HMNet), combining a high-resolution branch with multi-resolution branches for adaptive tumor feature tracking. HMNet incorporates a lightweight conditional channel weighting block and a Lightweight Multi-Resolution Feature Fusion (LMRF) module, reducing GPU load and model complexity, thereby optimizing segmentation efficiency. Liu et al. (15) presented an advanced lightweight 3D algorithm with an attention mechanism for brain tumor image segmentation based on the 3D-UNet architecture. Their approach includes hierarchical decoupled convolutions for parameter reduction, dilated convolutions for improved multiscale processing, and an attention mechanism at the output layer that focuses on tumor regions to improve segmentation accuracy. Zhao et al. (16) introduced a brain tumor segmentation method that integrated the complete convolutional neural network (FCNN) and the conditional random field (CRF) into a cohesive framework. The segmentation model was trained on the axial, coronal and sagittal planes of MRI images, which reduced the computational cost of 3D CNN while maintaining segmentation accuracy. Kong et al. (17) proposed a hybrid pyramid U-network that extracts multi-scale information using a downsampling path, an upsampling path, and a hybrid pyramid path. The combination of multi-scale, semantic, and location information improved the segmentation performance of the model. Finally, Bal et al. (18) presented a deep learning-based model with three different CNN architectures and manual features for multi-classification of brain tumors, which simplified the identification of the core and enhanced the prominent boundary of the tumor region, thereby improving the segmentation performance.

### 2.3 Our work

Brain tumor segmentation is an important aspect of medical image analysis that involves distinguishing tumor regions from surrounding healthy brain tissue. Accurate segmentation is critical for diagnosis, treatment planning, and monitoring of disease progression. Despite significant advances in the use of deep learning frameworks for brain tumor segmentation, achieving accurate segmentation remains challenging due to several factors (19). Tumor heterogeneity is a primary obstacle in brain tumor segmentation, as tumor regions exhibit different properties, including texture, intensity, and shape. Furthermore, the complexity of tumor shape, which can be irregular and asymmetric, poses an additional challenge (20).

To address these challenges, we propose an innovative continuous learning framework for brain tumor segmentation that integrates multi-scale spatial distillation and pseudo-labeling strategies. Our proposed framework aims to overcome the obstacles of brain tumor segmentation and achieve accurate segmentation results. The proposed model employs four parallel ResNet18 and DeepLabV3+ network architectures, which enhances the model’s feature extraction capabilities of the model while reducing the number of model parameters. This configuration allows the model to extract the most representative and discriminative features of brain tumor regions for accurate segmentation. To address the issues of catastrophic forgetting and unbalanced data categories during model training, we implement a multi-scale spatial distillation scheme and a confidence-based pseudo-labeling technique. The proposed method has been rigorously evaluated on three publicly available datasets and one private dataset, and shows competitive performance compared to other state-of-the-art segmentation techniques. The main innovations of this approach can be summarized as follows:

1. The introduction of a multi-scale spatial distillation scheme, specifically designed to effectively preserve knowledge during the continuous learning process. This method preserves both long- and short-range spatial relationships, mitigating the problem of catastrophic forgetting and promoting robust model performance.
2. The incorporation of a confidence-based pseudo-labeling technique that allows the model to recognize previously learned classes associated with current non-tumor tissue (background) pixels, overcoming potential non-tumor tissue (background) shift challenges and ensuring accurate segmentation results.
3. The use of four parallel ResNet18 and DeepLabV3+ network architectures to enhance the model’s feature extraction capabilities while reducing the number of model parameters. This arrangement ultimately results in improved segmentation accuracy and overall model efficiency.

The structure of this paper is as follows: In Section III, we provide a comprehensive overview of the study and introduce our proposed model. In Section IV, we describe the datasets, preprocessing techniques and evaluation metrics used in our investigation, as well as the experimental details. Section V presents the experimental results and discusses their implications for addressing the research question at hand. Section VI provides an in-depth discussion of our proposed method and elaborates on its contributions to the field of medical image analysis. Finally, Section VII concludes the paper with a synthesis of the main findings and suggests directions for future research in this area.

## 3 Methodology

### 3.1 Network architecture

**Figure 1** illustrates the proposed framework, which includes four parallel ResNet18 and DeepLabV3+ network architectures. Part of the segmentation model consists of four parallel ResNet18 structures that operate simultaneously to extract features from the input data. This arrangement efficiently enhances the model’s ability to capture diverse features, as each ResNet18 structure learns different representations of the input data. In addition, using multiple ResNet18 architectures in parallel reduces the total number of parameters required compared to using a single deeper network. This results in a more efficient and less computationally intensive model, while maintaining robust feature extraction capabilities.

**Figure 2:**
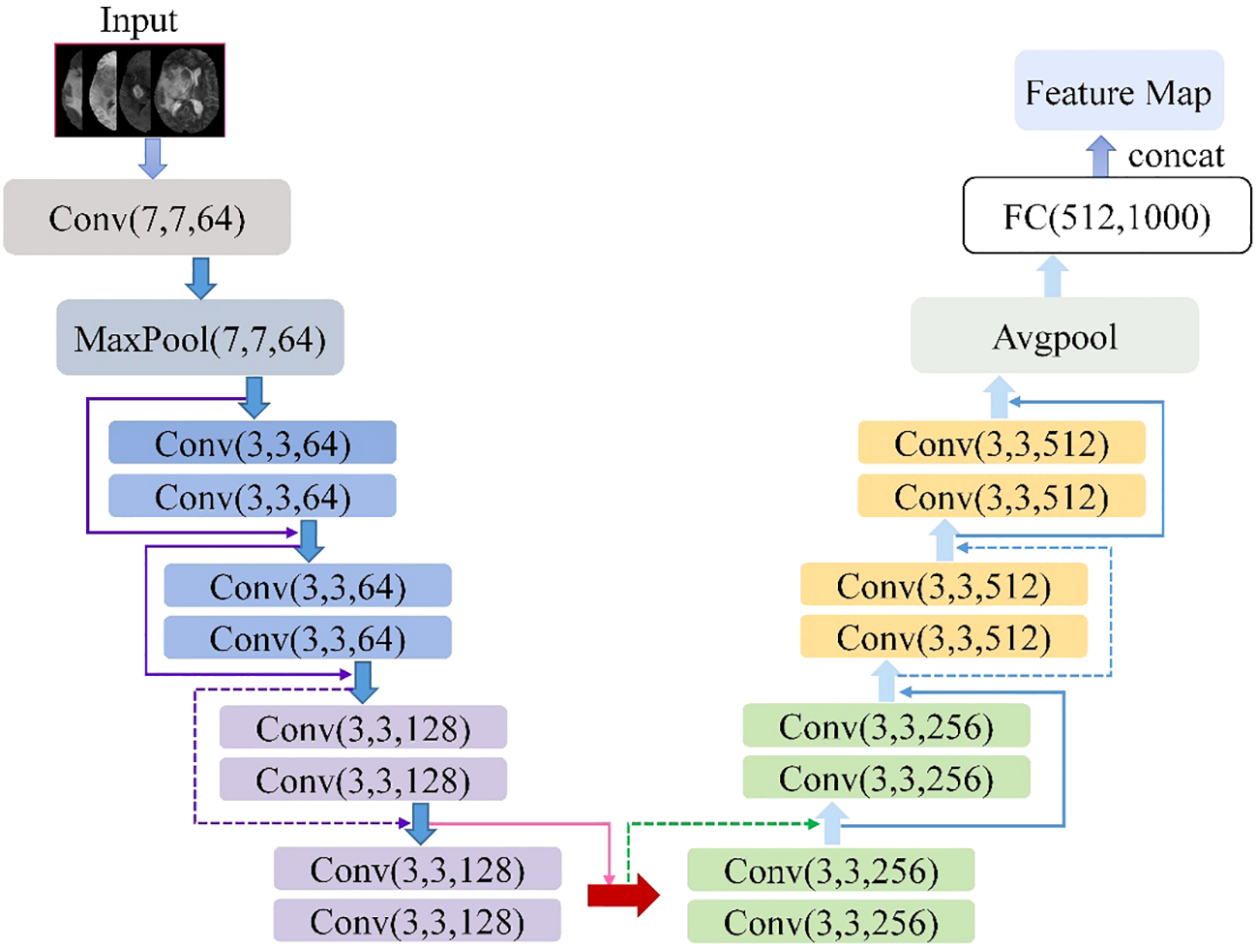
Architecture schematic of ResNet18.

The ResNet18 is a variant of the ResNet family introduced by He et al. (21). **Figure 2** shows the ResNet18 architecture, which is structured as follows: a 7×7 convolutional layer with 64 filters and a step size of 2; a 3×3 max-pooling layer with a step size of 2; four blocks, each containing two residual units with 64, 128, 256, and 512 filters, respectively; a global average pooling layer; a fully connected layer with the desired number of output classes. It is observed that the ResNet network utilizes a deep residual learning framework to address the degradation problem. Using a set of convolutional kernels, the network effectively extracts relevant image features from four MRI modalities, specifically FLAIR, T1, T1-CE and T2. In addition, the residual connections between the layers allow for faster forward propagation within the network.

**Figure 3:**
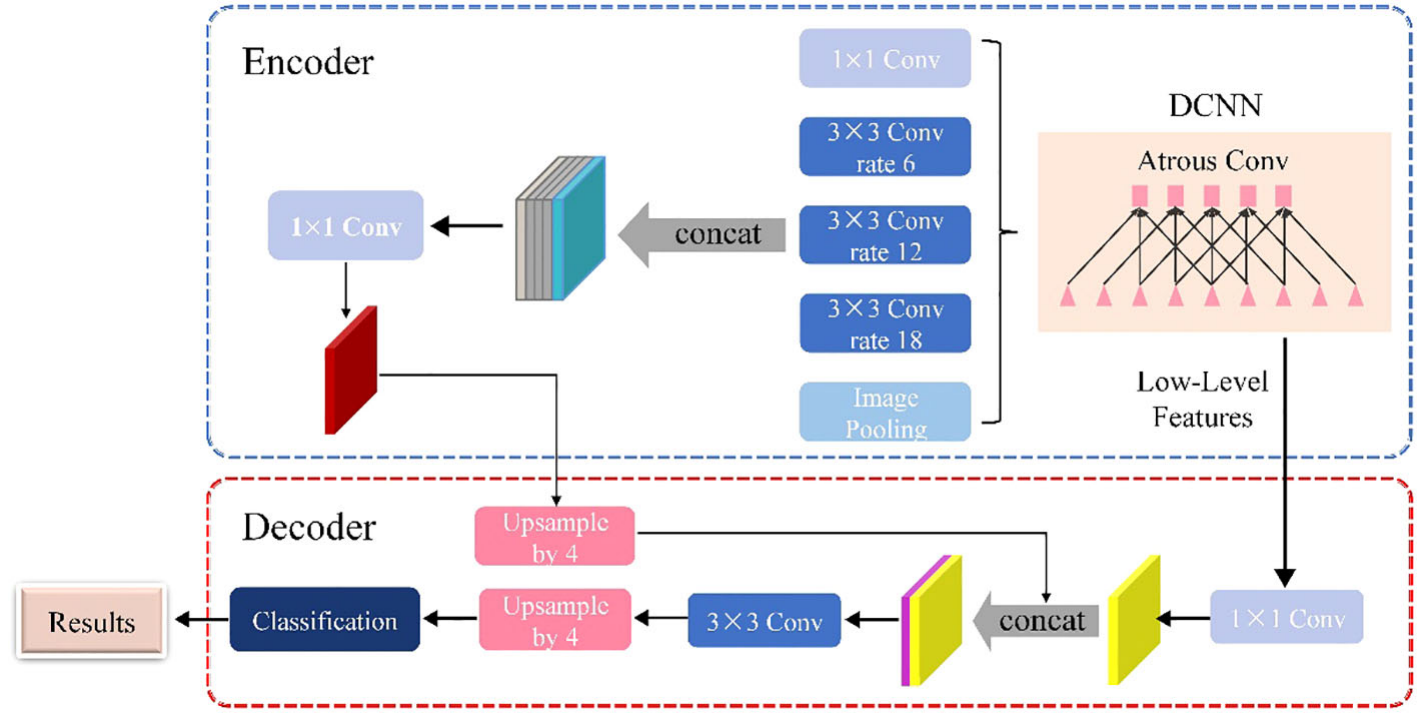
Schematic of the DeepLabV3+ architecture.

As shown in **Figure 3**, the DeepLabV3+ network is basically divided into two main components: an encoder and a decoder. The DeepLabV3+ network structure integrates multiple dilated (atrous) convolutions within the encoder segment, effectively enlarging the receptive field without losing information. As a result, each convolution captures a wider range of information. The encoder uses atrous spatial pyramid pooling (ASPP), where atrous convolutions with different dilation rates are applied in parallel to extract features individually. These extracted features are then merged and convolved to efficiently compress and consolidate the information.

In the decoder section, the initially compressed efficient feature layer, which has undergone double compression, is convolved to change the number of channels. This adjusted layer is then fused with the results of the efficient feature layer after applying the dilated convolution. This fusion process integrates low-level features with high-level features, improving the accuracy of segmentation boundaries and capturing finer details. Upon completion of the stacking process, two depth-separable convolution blocks are implemented. These blocks are designed to reduce computational complexity while maintaining the ability to learn complex features. The decoder module refines the segmentation results along object boundaries in a simple yet effective manner. This refinement produces a more accurate map of segmentation results, which ultimately helps to accurately identify and delineate target objects within the input image.

### 3.2 Multi-scale local spatial distillation scheme

In machine learning, continuous learning models often face the problem of catastrophic forgetting. This phenomenon occurs when new tasks are trained using the back-propagation deep learning technique, culminating in a significant decline in the model’s performance on previously acquired tasks. To address this dilemma, a common strategy involves the use of distillation loss, which establishes a balance between strict and relaxed constraints for sustainable learning (22). In this study, we propose a local pooling distillation (POD) scheme as an improved method to mitigate catastrophic forgetting, building on the approach proposed by Douillard et al. (23).

According to previous research, POD matches global statistics at different feature levels between the old and current models (23). We divide the dataset into *D_t_*, *t*=1,2…*T* according to the mask category, and *t* represents the required segmentation area. Each dataset *D_t_* consists of a group of (*I_t_,M_t_*), the former representing the input image with the size of *W* × *H*, and the latter representing the corresponding ground truth mask. Among them, *M_t_* only includes the tags of the current category *C ^t^*. Let *x* be the embedded tensor with the size of *H* × *W* × *C*, Ф*^t^* be the set of learnable parameters of the current network, f(·) be the encoder, and g(·) be the decoder. Extracting the POD embedded Ф consists in concatenating the *H* × *C* width-pooled slices and the *W* × *C* height-pooled slices of *x*, as shown in Equation 1:


(1) $$\Phi (x)=\left[\frac{1}{W}\sum_{\omega =1}^{W}x[:,\omega ,:]\|\frac{1}{H}\sum_{h=1}^{H}x[h,:,:]\right]\in R^{\left(H+W\right)\times C}$$

Where [·‖·] represents the series on the channel axis. For both the old and the current model, we compute embeddings at multiple levels. As shown in Equation 2, the goal of the POD loss is to minimise the L2 distance between these two sets of embeddings using the current network parameters Ф*^t^*:


(2) $$L_{pod}\left(\Theta^{t}\right)=\frac{1}{L}\sum_{l=1}^{L}{\left\|\Phi \left(f_{l}^{t}\left(I\right)\right)-\Phi \left(f_{l}^{t-1}\left(I\right)\right)\right\|}^{2}$$

In a continuous learning environment, where a model is incrementally trained on new data without forgetting previously learned knowledge, achieving high performance in both classification and segmentation tasks is essential. In classification, a global pooling operation is commonly used to aggregate features from the entire input and produce a fixed-length representation. However, this operation discards spatial information, which is critical for segmentation tasks that require high spatial accuracy.

On the other hand, segmentation requires the model to predict the class label for each pixel in the input image, and accurate localization of small objects is critical for high performance. However, modeling the entire width or height statistics of the input can obscure important local statistics of smaller objects, resulting in poor segmentation performance.

Therefore, in a continuous learning environment, distillation methods that transfer knowledge from a previously learned model to a new model should preserve the spatial relationship between long- and short-range information to achieve the most advanced results. This means that the distillation method should not only transfer global features, but also take into account the local spatial context. By preserving the spatial relationship between long-range and short-range information, the model can achieve high performance in both classification and segmentation tasks.

In this study, we introduce a new local POD feature extraction scheme. It involves the calculation of width and height merged slices over multiple regions extracted at different scales. For the embedding tensor *x* with size *H* × *W* × *C*, when the scale is 1/2*^s^*, the local POD embedding Ψ*^s^* (*x*) at the scale is calculated in Equations 3 as a series of *s* ^2^POD embeddings:


(3) $$\Psi^{s}\left(x\right)=\left[\Phi \left(x_{0,0}^{s}\right)\left\|\cdots \right\|\Phi \left(x_{s-1,s-1}^{s}\right)\in R^{\left(H+W\right)\times C}\right]$$

Where ∀i=0⋯s−1 , ∀i=0⋯s−1 , $x_{i,j}^{s}=x\left[iH/s:\left(i+1\right)H/s,jW/s:\left(j+1\right)W/s,:\right]$ is a sub-region of the embedded tensor *x*, with the size of *W*/*s* × *H*/*s*. Connect the local POD of each scale *s* along the channel axis and embed Ψ*^s^* (*x*) to form the final embedding in Equation 4:


(4) $$\Psi \left(x\right)=\left[\Psi^{1}\left(x\right)\left\|\cdots \right\|\Psi^{S}\left(x\right)\right]\in R^{\left(H+W\right)\times C\times S}$$

For several layers of the old model and the current model, we compute the local POD embeddings. The final local loss of the POD is shown in (Equation 5):


(5) $$L_{Local\text{POD}}\left(\Theta^{t}\right)=\frac{1}{L}\sum_{l=1}^{L}{\left\|\Psi \left(f_{l}^{t}\left(I\right)\right)-\Psi \left(f_{l}^{t-1}\left(I\right)\right)\right\|}^{2}$$

Local POD preserves both long-range and short-range spatial relationships, which is critical for reducing catastrophic forgetting in a continuous learning environment. Catastrophic forgetting occurs when a model loses previously learned knowledge when trained on new data. In a continuous learning setting, this can lead to a significant drop in performance on previously learned tasks. To address this problem, the Local POD method distils knowledge from a previously learned model into a new model by preserving the spatial relationship between long-range and short-range information. This allows the model to retain previously learned knowledge while learning new information.

### 3.3 Confidence-based pseudo-labeling strategy

In a continuous learning environment, labeling pixels as non-tumor tissue (background) can be challenging because these pixels can belong to either old or future classes. Treating these pixels as non-tumor tissue (background) can lead to catastrophic forgetting, where the model forgets previously learned knowledge. To address this issue, we propose a pseudo-labeling strategy for background pixels. Pseudo-labeling is a common technique used in domain adaptation for semantic segmentation, where a model is trained on a combination of real labels from a source dataset and pseudo-labels assigned to an unlabeled target dataset (24). In our case, we use predictions from the previously learned model for background pixels as cues to their real class, especially if they belong to one of the old classes. By using these predictions as pseudo-labels, we can more accurately label background pixels and avoid catastrophic forgetting.

Formally, let *C ^t^* = *card* (*C ^t^*) - 1 represent the cardinality of the current classes (excluding the background class). Let ${\hat{S}}^{t}\in R^{W,H,1+C^{1}+\cdots +C^{t}}$ represent the predictions of the current model (including the real background class, all the old classes, and the current classes). Let ${\tilde{S}}^{t}\in R^{W,H,1+C^{t}}$ take the target as step *t*, and compute using the one-hot ground-truth segmentation map at step *t* and the pseudo tags extracted by using the old model predictions, as shown in (Equation 6) below:


(6) $${\tilde{S}}^{t}(w,h,c)=\left\{ \begin{array}{l} 1\text{ if }S^{t}(\omega ,h,c_{bg})=0\text{ and }c=\underset{c^{\prime }\in C^{t}}{\arg\max S^{t}}\left(\omega ,h,c^{\prime }\right)\\ 1\text{ if }S^{t}(\omega ,h,c_{bg})=1\text{ and }c=\underset{c^{\prime }\in C^{1:t-1}}{\arg\max{\hat{S}}^{t-1}}\left(\omega ,h,c^{\prime }\right)\\ 0\text{ otherwise} \end{array}\right.$$

For non-background pixels, since these pixels are associated with known classes, we use the ground truth label as the true label. Otherwise, we use the class predicted by the old model $g^{t-1}\left(f^{t-1}\left(\cdot \right)\right)$ . However, for uncertain pixels where the old model may fail, pseudo-labelling all background pixels may be invalid. Therefore, only pseudo-labels with sufficient “confidence” of the old model are retained. To account for this uncertainty, the equation is modified as follows (Equation 7):


(7) $${\tilde{S}}^{t}(w,h,c)=\left\{ \begin{array}{l} 1\text{ if }S^{t}(\omega ,h,c_{bg})=0\text{ and }c=\underset{c^{\prime }\in C^{t}}{\arg\max S^{t}}\left(\omega ,h,c^{\prime }\right)\\ 1\text{ if }S^{t}(\omega ,h,c_{bg})=1\text{ and }c=\underset{c^{\prime }\in C^{1:t-1}}{\arg\max{\hat{S}}^{t-1}}\left(\omega ,h,c^{\prime }\right)\text{ and }u<\tau_{c}\\ 0\text{ otherwise} \end{array}\right.$$

Where *u* is the uncertainty of the pixels (ω,h) and *τ_c_* is a category specific threshold. Therefore, we discard all the pixels for which the old model is uncertain (*u* ≥ *τ_c_*) in (7) and decrement the normalisation factor WH by one. We use entropy as a measurement of uncertainty *u*. Specifically, before the learning task *t*, we compute the median entropy for the old model, which covers all pixels ϵ *C ^l:t^* ^-1^c of *D ^t^* predicted as class *c* by all previous classes *c*, and provides the threshold *τ_c_* ϵ *C^1:t^* ^-1^c. Referring to Saporta et al. (25), the pseudo-labelled cross-entropy loss of the old class can be written as (Equation 8):


(8) $$L_{pseudo}\left(\Theta^{t}\right)=-\frac{v}{WH}\sum_{w,h}^{W,H}\sum_{c\in C^{t}}\tilde{S}\left(\omega ,h,c\right)\log{\hat{S}}^{t}\left(\omega ,h,c\right)$$

Where *v* is the ratio of acceptable old class pixels to the total number of such pixels. The importance of pseudo-tags is adaptively weighted in the total loss. This method not only uses local POD to avoid catastrophic forgetting, but also uses uncertainty-based pseudo tags to solve for background offset. In summary, the total loss is given by (Equation 9):


(9) $$L\left(\Theta^{t}\right)=\underset{classification}{\underset{︸}{L_{pseudo}\left(\Theta^{t}\right)}}+\lambda \underset{classification}{\underset{︸}{L_{local\text{POD}}\left(\Theta^{t}\right)}}$$

Where *λ* is a super parameter.

## 4 Experimental setup

### 4.1 Dataset

In this study, three publicly available datasets and one private dataset are used to demonstrate the effectiveness and robustness of the proposed brain tumor segmentation method. The datasets used are the Multimodal Brain Tumor Segmentation Challenge 2019 (BraTS2019), the Multimodal Brain Tumor Segmentation Challenge 2020 (BraTS2020), the Multimodal Brain Tumor Segmentation Challenge 2021 (BraTS2021), and a proprietary dataset from the First Affiliated Hospital of Zhengzhou University (BraTS_FAHZU). BraTS2019 contains 335 training cases and 125 validation cases, while BraTS2020 expands the training set to 369 cases and maintains the same validation set as BraTS2019. BraTS2021 further expands the training set to 1, 251 cases and the validation set to 219 cases. The BraTS_FAHZU dataset, provided by the First Affiliated Hospital of Zhengzhou University, contains 232 patient cases manually annotated by two experienced radiologists.

The BraTS datasets are widely used in research for the development and evaluation of brain tumor segmentation algorithms due to the diversity of tumor types, sizes and locations that contribute to their challenging nature. These datasets include brain tumor MRI scans from four imaging modalities: T1, T1-CE, T2 and FLAIR, presented in the standard NIFTI format for storing and sharing medical imaging data. Contestants using these datasets are challenged to develop algorithms that accurately segment three sub-regions of brain tumors: enhancing tumor (ET), whole tumor (WT) and tumor core (TC).

### 4.2 Data preprocessing

**Figure 4:**
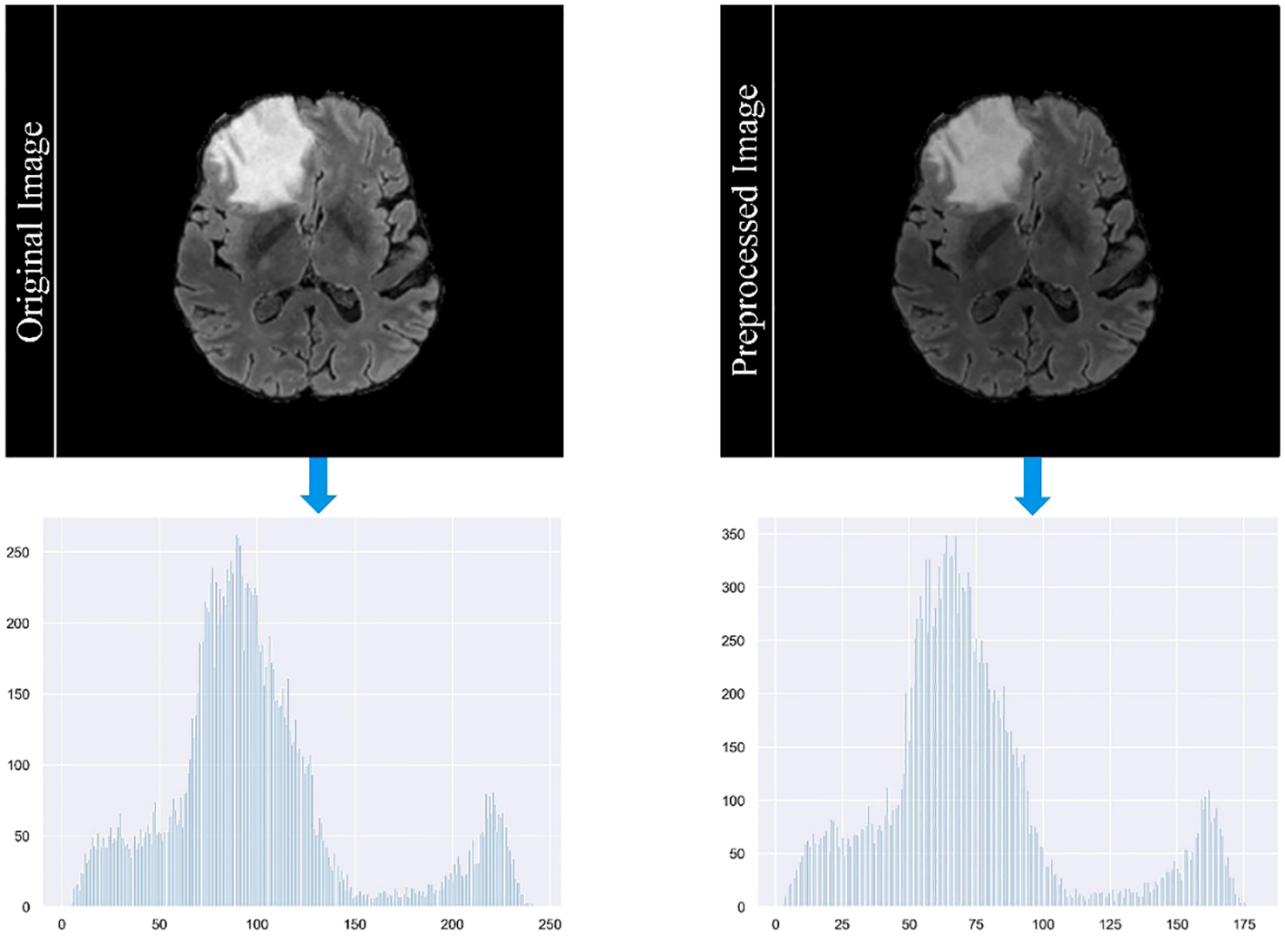
Comparison of MRI images before and after the standardization process.


In our study, we employ a careful data preprocessing protocol to ensure the accuracy and reliability of our brain tumor segmentation model. This process is critical for handling the diverse range of MRI modalities present in our datasets, each characterized by unique image contrasts. The variance in these contrasts can lead to the problem of gradient vanishing during model training, a phenomenon in which gradient updates become negligible, hindering learning efficiency. To counteract this, we implemented the z-score standardization method across all modalities. Each MRI scan is standardized by subtracting the mean and dividing by the standard deviation of its intensity values. This method normalizes the intensity distribution across scans and ensures a consistent scale. As a result, it reduces the impact of outliers and improves the ability of the model to discriminate relevant features. **Figure 4** in our manuscript provides a visual comparison of MRI images before and after this standardization process, illustrating the improved clarity and discriminability of tumor features in the standardized images. The formula for calculating the *z*-score of a data point *x* in a population with mean *μ* and standard deviation *σ* is given in Equation 10:

(10) $$z=\frac{x-\mu }{\sigma }$$

Recognizing the prevalence of non-tumor tissue in most MRI scans, we implement a data cropping strategy to focus on the regions of interest. We crop images to a standardized size of 160×160×160 pixels, specifically retaining those that contained labeled tumor regions. This approach minimizes the model’s exposure to the vast majority of non-tumor tissue, which could otherwise bias its learning process toward these areas. Although this method may result in some loss of peripheral information, it has been empirically shown to improve the performance of deep learning models in segmenting small and isolated tumor regions.

In addition to the above steps, we use data enrichment techniques to further increase the robustness of our dataset. This includes data augmentation methods such as rotation, flipping, and scaling to increase the variability and volume of the training data. Such augmentation helps to simulate different scenarios and orientations of brain tumors, thus enabling the model to better generalize across different cases. It’s important to note that these augmentations were carefully calibrated to preserve the realistic anatomical structures and characteristics of brain tumors.

The meticulousness of our data preprocessing protocol plays a critical role in the performance of our segmentation model. These steps, from standardization to data enrichment, are designed to address the specific challenges posed by the variability of MRI data and the nature of brain tumors. The comprehensive description of these methods in our manuscript aims to ensure reproducibility, a fundamental aspect of scientific research.

### 4.3 Evaluation metrics

In order to thoroughly evaluate the effectiveness of our proposed framework for segmentation tasks in various domains, including medical imaging and computer vision, we use four widely accepted evaluation metrics. These metrics include the Dice coefficient (Dice), sensitivity, specificity, and Hausdorff95 distance (Haus95), which are explicitly defined in (Equations 11–14), respectively. The Dice quantifies the similarity between two sets, such as ground truth (GT) and predicted segmentation masks, with a higher value indicating a better match between them. Sensitivity represents the ability of the algorithm to accurately detect positive instances, while specificity represents its ability to correctly identify negative instances. A higher value for sensitivity or specificity indicates better performance in detecting positive or negative instances, respectively. In addition, the Haus95 calculates the maximum distance between a point in one set and its nearest point in the other set, providing a measure of the worst-case boundary discrepancy between the ground truth and the predicted masks. A lower Haus95 value indicates a better match between the two sets. Taken together, these four evaluation metrics provide a rigorous and comprehensive assessment of the segmentation performance of our proposed framework, allowing us to compare it with other segmentation algorithms and determine the most appropriate one for a given task.


(11) $$\text{Dice}=\frac{2\text{TP}}{\text{FN}+\text{FP}+2\text{TP}}$$

(12) $$\text{Sensitivity}=\frac{\text{TP}}{\text{TP+FN}}$$

(13) $$\text{Specificity}=\frac{\text{TN}}{\text{TN+FP}}$$

(14) $$\mathrm{Haus95}(T,P)=\max\{\underset{t\in T,p\in P}{\sup\inf}d(t,p),\underset{p\in P,t\in T}{\sup\inf}d(t,p)\}$$

Where TP, TN, FP, and FN are the numbers of true positives, true negatives, false positives, and false negatives respectively, sup denotes the supremum and inf denotes the infimum, *t* and *p* donate the points on the surface *T* of the GT region and the surface *P* of the predicted region, d(·,·) is a function of the distance between the point *t* and *p*.

### 4.4 Experimental details

The training process for deep learning models requires careful tuning of many hyperparameters, including learning rate and epoch count, as these can have a significant impact on the performance of the model. To achieve optimal results, these hyperparameters must be configured appropriately. Identifying the ideal values for these hyperparameters typically involves an empirical procedure in which different values are explored and their impact on the model’s performance is evaluated. In our study, we set the initial learning rate at 0.0325, a common value for segmentation tasks. We used the Poly strategy to dynamically modify the learning rate, which systematically decreases it over successive iterations. This method effectively reduces overfitting and improves the generalization ability of the model. In addition, we assigned a momentum value of 0.9, a common choice in deep learning. This parameter speeds up the learning process by maintaining a moving average of the gradients. We trained our model for approximately 30 epochs, which is an appropriate duration for image segmentation tasks because it gives the model enough time to learn relevant features and converge to an optimal solution. Finally, we randomized the order of the data sets during training to increase the robustness of the training process. This technique prevents the model from memorizing the order of the data sets, which can contribute to overfitting and undermine the model’s ability to generalize.

Our experiments were conducted in an environment using TensorFlow 1.13.1 and Python 3.6.5, with PyCharm as the integrated development environment (IDE). The hardware configuration included an Intel(R) Xeon(R) Silver 4210 CPU running at 2.20 GHz, a 64-bit Windows 10 operating system, 32 GB of RAM, and two graphics cards: an ASPEED graphics family (WDDM) and an NVIDIA TITAN RTX. This setup provided a robust foundation for efficiently executing deep learning tasks and ensuring reliable results.

## 5 Results

### 5.1 Ablation study

The ablation study detailed in **Table 1** is carefully designed to identify the most efficient and accurate network architecture for brain tumor segmentation. Our selection of ResNet18 in combination with DeepLabV3 is validated by the highest Dice scores achieved on BraTS2020 training set, supporting the premise that the streamlined architecture of ResNet18 is adequate for robust feature extraction without the computational cost associated with the more complex ResNet50. The fusion of ResNet18 with the contextual knowledge of DeepLabV3 results in a model adept at segmenting both the whole tumor and its core, demonstrating the strength of this combination in capturing essential tumor details while maintaining computational economy. The reduced Dice scores associated with the deeper ResNet50 raise concerns that additional depth may not be beneficial for brain tumor segmentation, possibly due to complications such as overfitting, which can degrade model performance. In contrast, the UNet architecture, while commendable in its performance, does not appear to exploit the full potential of the representational depth offered by the ResNet backbones as effectively as DeepLabV3, underscoring the critical need for an architecture that can capitalize on the depth of extracted features for accurate segmentation.

**Table 1:** Comparison of ablation results for different combinations of backbone networks.

Combination	Dice_ET	Dice_WT	Dice_TC
**ResNet18+DeepLabV3**	0.673	0.872	0.741
**ResNet50+DeepLabV3**	0.637	0.858	0.726
**ResNet18+Unet**	0.613	0.843	0.705
**ResNet50+Unet**	0.602	0.824	0.698

**Figure 5:**
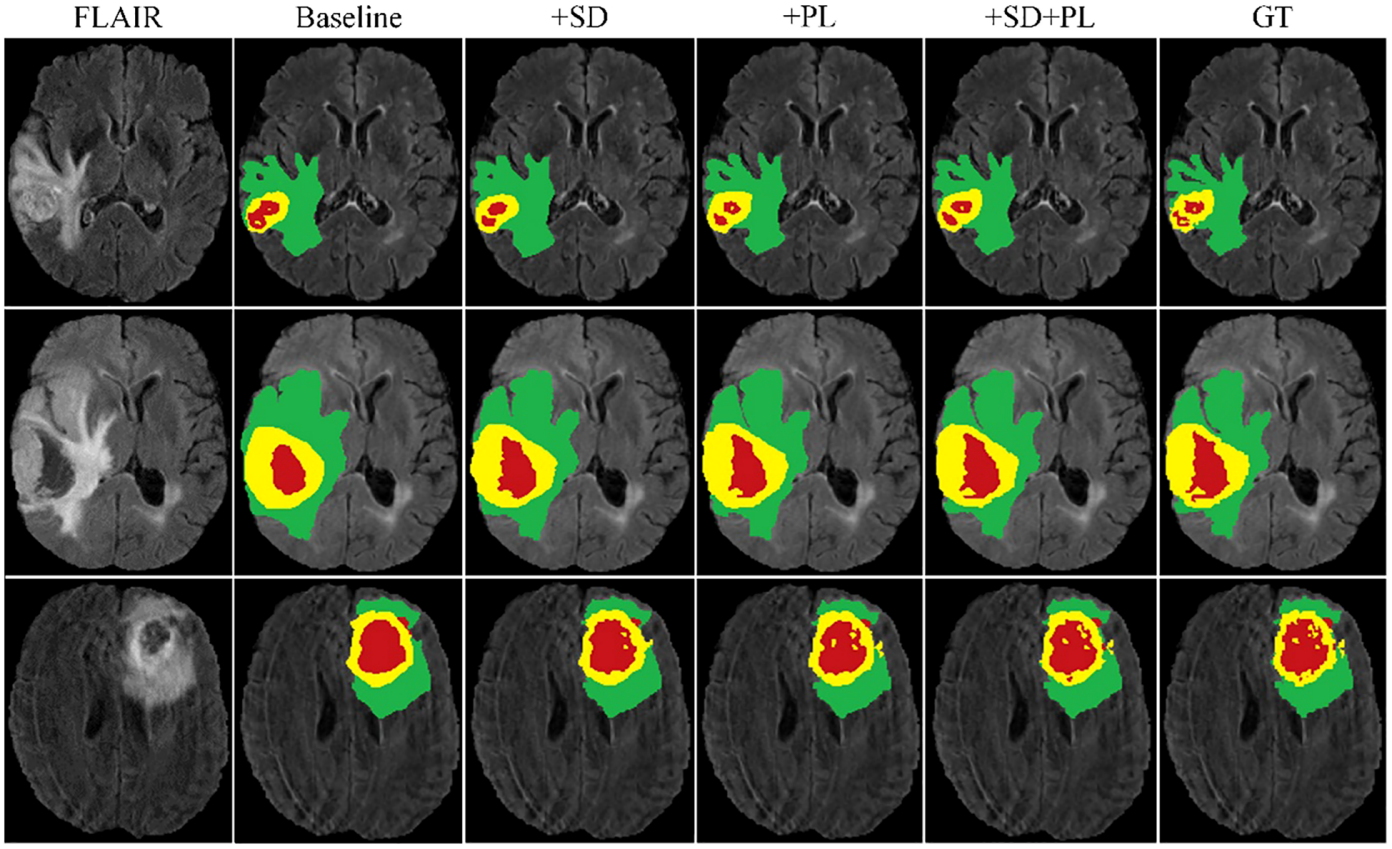
Cross-validation results on the BraTS2020 training set for baseline model comparison.

Building on this foundational analysis, our study extends into the areas of multi-scale spatial distillation and confidence-based pseudo-labeling to enhance segmentation accuracy. Using the BraTS2020 training set and a robust 10-fold cross-validation protocol, we computed the mean results shown in **Table 2**. Here we quantify the effectiveness of our model, while **Figure 5** provides a visual demonstration of its segmentation capabilities. Our research has evolved into a sophisticated continuous learning framework that integrates the core strengths of ResNet18 and DeepLabV3+ with spatial distillation (SD) and confidence-based pseudo-labeling (PL). This complex synthesis, SD+PL, represents the pinnacle of our model’s capabilities and provides a nuanced and systematic examination of its performance. The reported results not only validate the model’s operational merits, but also its adaptability and precision, making it a promising solution for complex segmentation tasks in medical imaging.

**Table 2:** Performance metrics of proposed continuous learning network components through 10-fold cross-validation on the BraTS2020 training set.

Component	Dice			Sensitivity			Specificity			Haus95		
	ET	WT	TC	ET	WT	TC	ET	WT	TC	ET	WT	TC
**Baseline**	0.673	0.872	0.741	0.682	0.861	0.726	1.000	0.999	0.999	45.8	12.14	15.6
**+SD**	0.697	0.873	0.775	0.716	0.859	0.733	1.000	1.000	1.000	39.7	15.03	20.2
+PL	0.729	0.881	0.823	0.757	0.887	0.838	0.999	0.996	0.998	34.9	10.64	11.3
+SD+PL	0.761	0.889	0.867	0.798	0.912	0.871	1.000	1.000	0.999	28.7	9.35	8.2

A careful analysis of **Table 1** shows that the hybrid of the baseline model and pseudo-labeling (denoted as ‘baseline+PL’) demonstrates superior performance over the baseline model integrated with spatial distillation (denoted as ‘baseline+SD’) for several accuracy indicators. Looking at the Dice scores, the ‘baseline+PL’ model yields values of 0.729, 0.881, and 0.823 for the enhancing tumor, the whole tumor, and the tumor core, in that order. These values are significantly better than their counterparts in the ‘baseline+SD’ model, which are 0.697, 0.873 and 0.775, respectively. This represents a performance improvement of 4.6% for the enhancing tumor, 0.9% for the total tumor, and 6.2% for the tumor core when using the ‘baseline+PL’ model.

When considering Hausdorff95 metrics, baseline+PL achieves values of 34.9 for the enhancing tumor and 11.3 for the tumor core. In contrast, the ‘baseline+SD’ model documents values of 39.7 and 20.2, illustrating a reduction in distance of 4.8 and 8.9 for the enhancing tumor and the tumor core, respectively, with the application of the ‘baseline+PL’ model.

The graph in **Figure 5** illustrates the ability of the enhanced model ‘baseline+PL’ to accurately segment individual regions. In particular, it avoids the misclassification of the gadolinium-enhanced (GD) tumor region located at the periphery of the tumor. Furthermore, the integration of the baseline model with spatial distillation and pseudo-labeling techniques (referred to as ‘baseline+SD+PL’) shows a significant increase in sensitivity and specificity compared to the ‘baseline+PL’ model. As shown in **Table 1**, the ‘baseline+SD+PL’ model demonstrates superior performance with an increase in sensitivity and specificity of 5.4% and 0.1% for the enhancing tumor, 3.9% and 0.1% for the core tumor, and 2.8% and 0.1% for the total tumor, respectively.

### 5.2 Results

**Figure 6:**
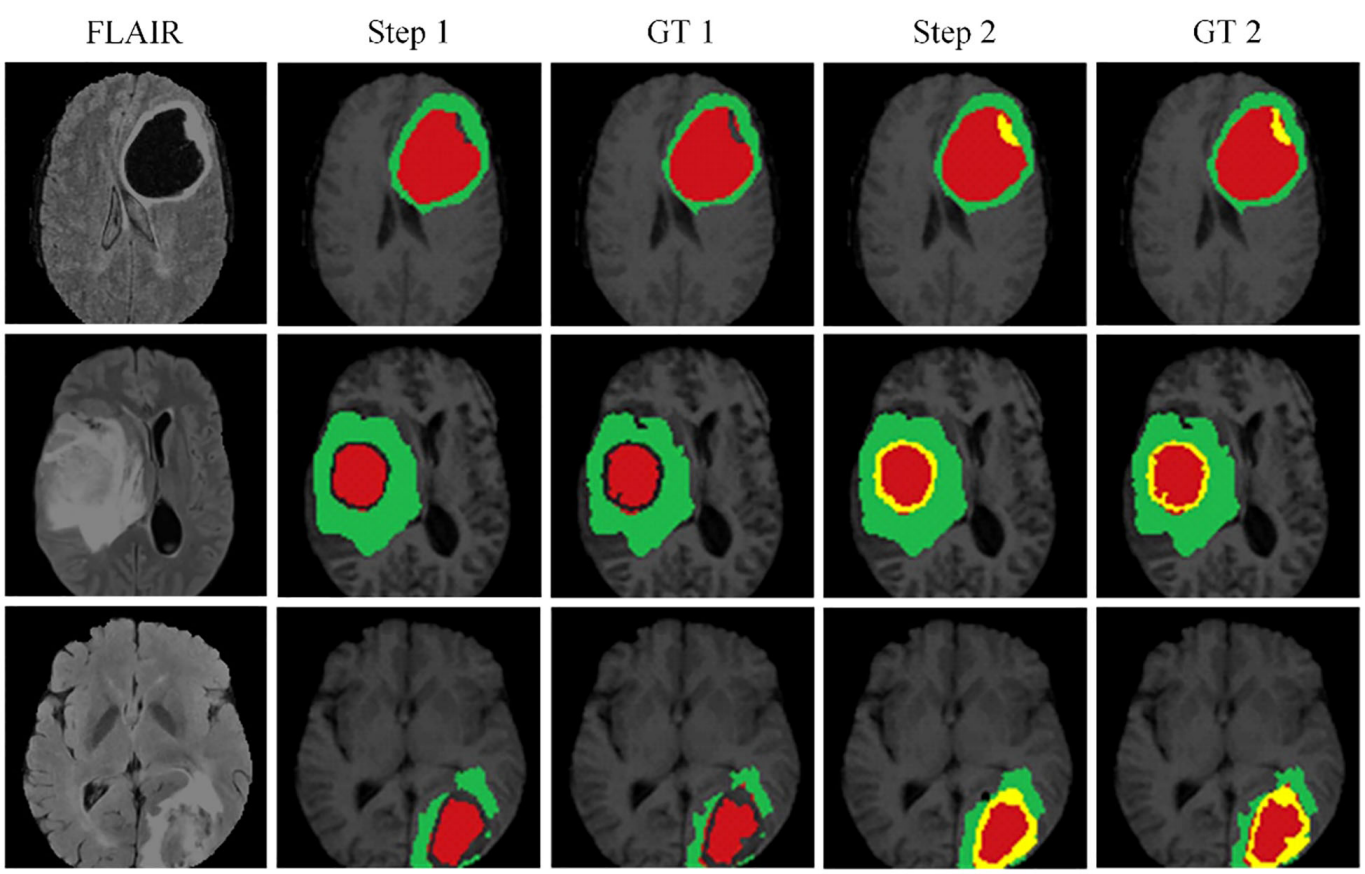
The proposed continuous learning network training dynamics demonstrate resistance to catastrophic forgetting.

**Figure 6** illustrates the effectiveness of our continuous learning model by demonstrating its ability to maintain accuracy in segmenting necrotic and edematous regions (step 1) while successfully integrating a new class-enhancing tumor regions (step 2)-without compromising the segmentation quality of previously learned classes. Comparison with the corresponding ground truths (GT 1 and GT 2) shows minimal deviation, indicating that our model effectively combats catastrophic forgetting. This visual evidence of the model’s sustained performance across learning phases justifies further development and application of our model in clinical settings where dynamic learning is critical.

To support the visual evidence, our research includes a rigorous evaluation framework where the model is trained on three separate and sequentially released brain tumor MRI datasets - BraTS2019, BraTS2020, and BraTS2021. Evaluation is performed on the corresponding validation sets to assess the model’s segmentation performance, accuracy, and ability to generalize across these temporally varying datasets. Furthermore, to ensure the robustness of the model and to evaluate its performance on unseen data, we also test the model on an independent, undisclosed dataset. The quantitative results of this exhaustive evaluation are systematically documented in **Tables 3**, **4**, which detail the performance metrics for each dataset. These metrics, which include but are not limited to Dice scores, precision, and recall, provide a comprehensive view of the model’s consistency and the effectiveness of its generalization capabilities, underscoring the model’s readiness for clinical adoption and its potential to contribute to the advancement of medical image analysis.

**Table 3:** Quantitative analysis of the proposed continuous learning network on the training sets of BraTS2019, BraTS2020 and BraTS2021.

data set	Dice			Sensitivity			Specificity			Haus95		
	ET	WT	TC	ET	WT	TC	ET	WT	TC	ET	WT	TC
**2019**	0.801	0.843	0.836	0.857	0.892	0.815	0.999	0.999	0.999	27.6	10.20	15.1
**2020**	0.761	0.889	0.867	0.798	0.912	0.871	1.000	1.000	0.999	28.7	9.35	8.2
**2021**	0.823	0.898	0.881	0.877	0.916	0.893	0.999	0.999	0.997	19.4	8.68	9.8

**Table 4:** Model evaluation on validation sets of BraTS2019, BraTS2020 and BraTS2021.

data set	Dice			Sensitivity			Specificity			Haus95		
	ET	WT	TC	ET	WT	TC	ET	WT	TC	ET	WT	TC
**2019**	0.716	0.785	0.778	0.793	0.837	0.773	0.999	0.997	0.999	28.9	17.52	20.6
**2020**	0.752	0.879	0.817	0.778	0.871	0.812	0.996	1.000	0.999	29.7	14.78	13.8
**2021**	0.759	0.803	0.819	0.812	0.846	0.794	0.999	0.999	0.999	20.5	11.65	19.2

A critical observation from these results is the model’s ability to effectively segment each subregion of the brain tumor, which is a significant contribution given the complexity of the disease. Of particular note is the high specificity of the model, which is close to 1. This high specificity metric underscores the model’s ability to accurately predict negative cases, a key requirement for effective medical imaging analysis. Furthermore, the high specificity of the model also implies a commendable performance in predicting non-tumor tissue (background). In other words, the model demonstrates the ability to correctly identify regions of the image that do not contain tumors, a crucial factor in obtaining accurate segmentation results and, consequently, accurate diagnosis and treatment planning. Thus, the presented model demonstrates both high performance and broad applicability in the challenging field of brain tumor segmentation.

**Figure 7:**
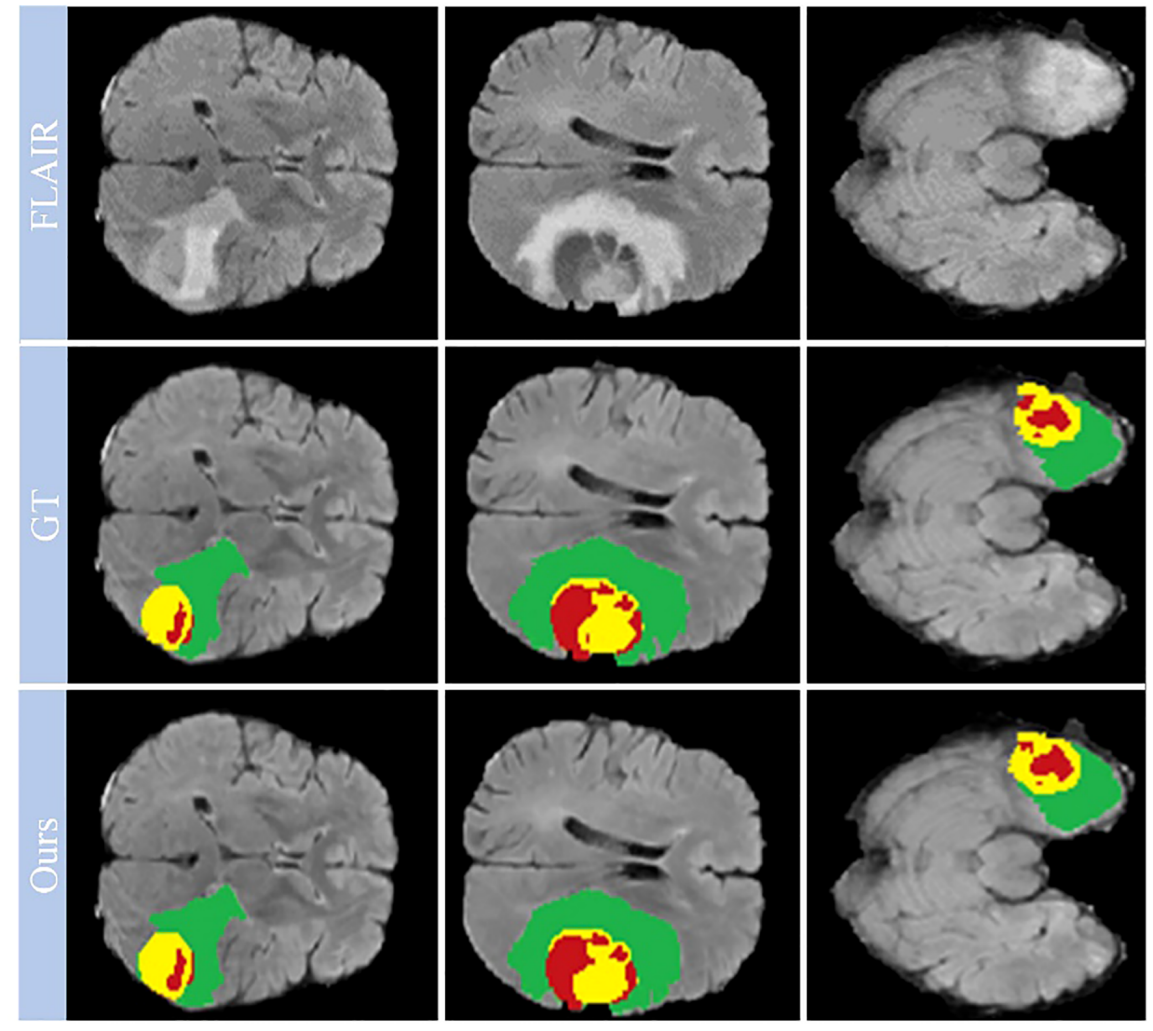
Qualitative performance results on the BraTS2019, BraTS2020 and BraTS2021 training datasets.

Looking closely at **Figure 7**, which shows selected examples from the training set, the model demonstrates an impressive ability to accurately segment brain tumors. This ability prevails across a spectrum of tumor characteristics, including different morphologies, different shapes, and different signal intensities. In addition, the model demonstrates a superior ability to delineate between tumor sub-regions, further enhancing its ability to provide high-fidelity representations of brain tumors. The ability to distinguish between these sub-regions has significant implications for the diagnosis and treatment of brain tumors, providing physicians with a more nuanced understanding of the disease presentation.

However, our observations reveal certain deviations from perfection. In particular, the boundaries of the ET region are areas where the model appears to stumble. The cause of these inaccuracies may be due to the increased signal intensity within this region, which may inadvertently cause the model to misclassify portions of the ET region as part of the TC region. This observation points to a critical area for future investigation and improvement, ultimately paving the way for a more reliable and accurate model for brain tumor segmentation.

**Figure 8:**
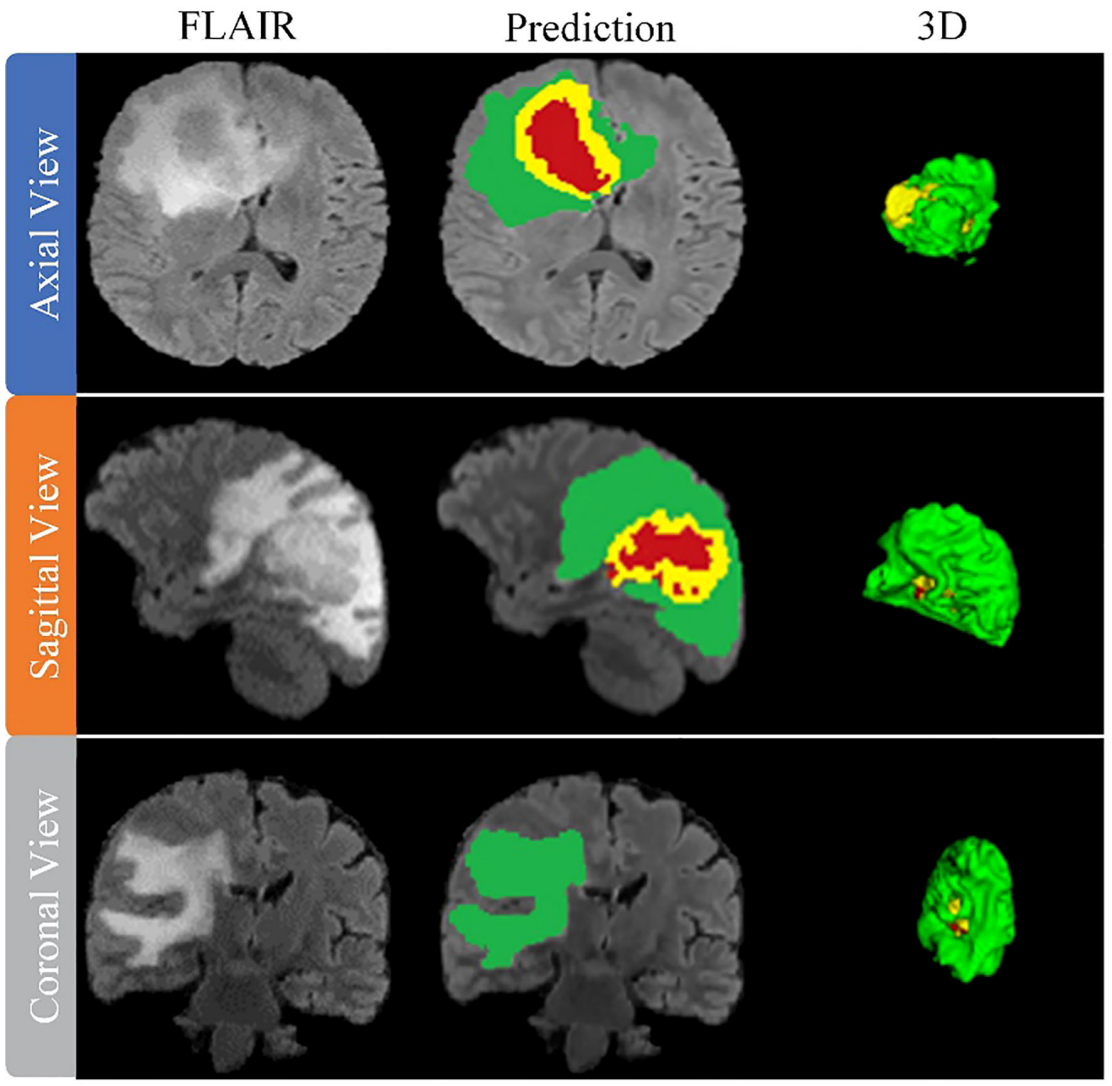
Performance evaluation on validation data sets from BraTS2019, BraTS2020 and BraTS2021.

After training the model, an arbitrary sample is selected from the validation set to illustrate its performance, as shown in **Figure 8**. This figure presents a series of MRI slices obtained from different perspectives, demonstrating the model’s ability to identify and delineate the regions of interest in all orientations when dealing with brain tumors. To further highlight the effectiveness of the model, we provide a 3D rendering of the segmentation results obtained from different viewpoints within the validation set. This robust 3D representation demonstrates the model’s ability to accurately identify and separate the sub-regions of the brain tumor, regardless of the viewing perspective. It reinforces the model’s exceptional ability to generate accurate segmentation across the entire MRI scan.

The model thus proves its worth by demonstrating robust performance in brain tumor segmentation, regardless of tumor characteristics such as size, shape, or signal intensity. Its robust performance and adaptability underscore its practical application in effectively mapping the intricate heterogeneity of gliomas, thus improving the accuracy of diagnosis and treatment planning in clinical settings.

The generalizability of our model is tested by applying it to the BraTS_FAHZU dataset, a collection of clinical data that has not undergone specific curation. We used 10-fold cross-validation, a well-established statistical technique that prevents overfitting and ensures an unbiased evaluation of the model’s application to unseen data. **Table 5** provides a numerical representation of the study results, suggesting the prospective competence of our model when applied to clinical datasets. This has implications for real-world healthcare implementations, suggesting a potential shift in the use of AI-driven solutions in practical settings.

**Table 5:** Quantitative results of the proposed continuous learning network on the BraTS_FAHZU private dataset.

Dice			Sensitivity			Specificity			Haus95		
**ET**	WT	TC	ET	WT	TC	ET	WT	TC	ET	WT	TC
**0.821**	0.847	0.872	0.853	0.919	0.867	0.996	0.999	0.998	20.6	9.79	15.7

**Figure 9:**
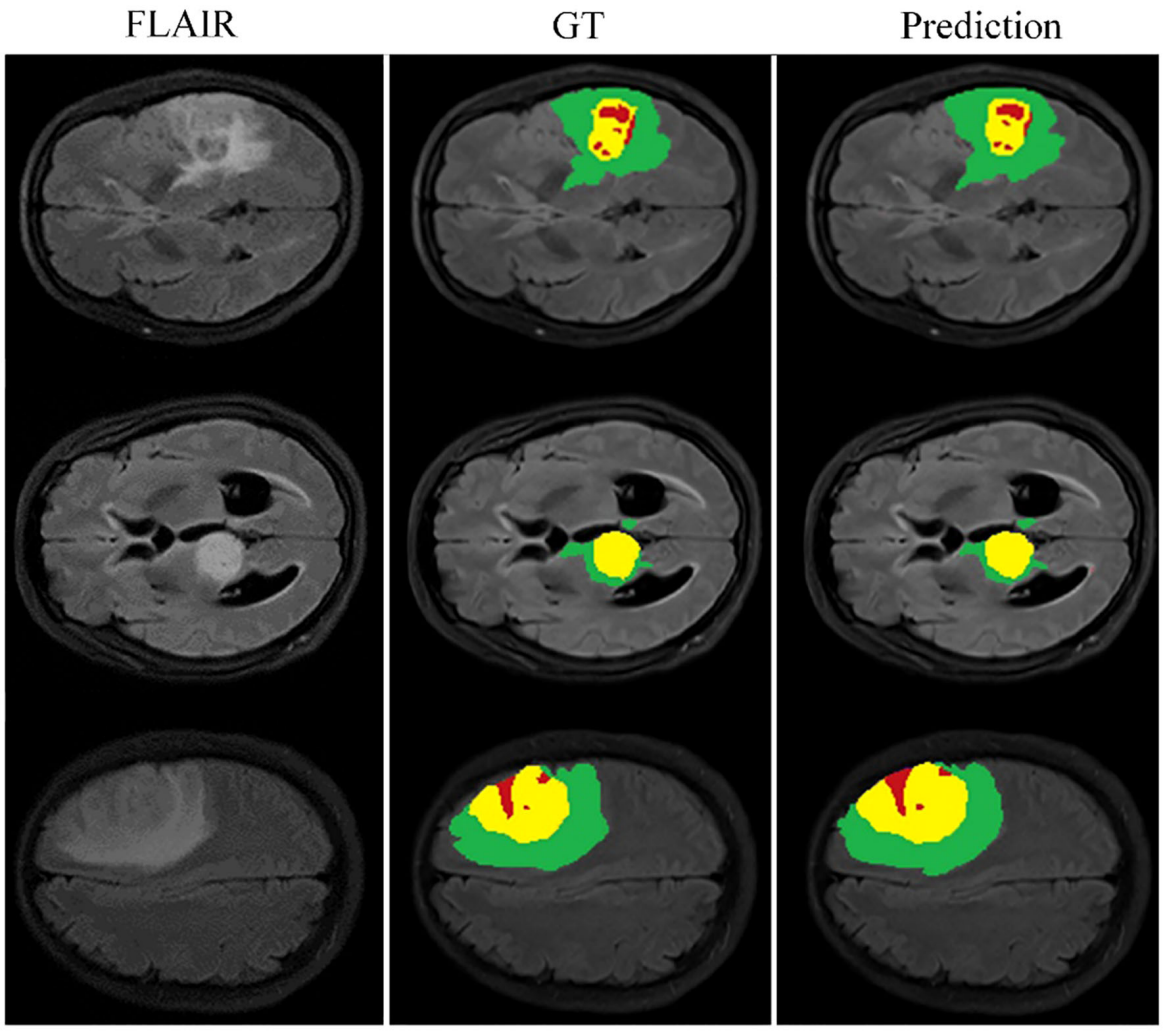
Model performance on the private dataset BraTS_FAHZU.

**Figure 9** also provides a visual representation of the model’s performance on the BraTS_FAHZU dataset, specifically with respect to brain tumor segmentation. The accuracy indicated by these results underscores the potential for the use of our model in clinical contexts, and highlights its validity in future diagnostic and treatment scenarios.

## 6 Discussion

In our study, we have carefully evaluated our proposed continuous learning network, a novel segmentation method, across three consecutive years of the Brain Tumor Segmentation (BraTS) challenge datasets-BraTS2019, BraTS2020, and BraTS2021. Our results, detailed in **Table 6** for the BraTS2019 dataset, highlight the proposed network’s dice score of 0.716 for enhancing tumors, which illustrates its strong ability to capture the intricate details of tumor morphology, although it does not surpass the performance of Minh et al. and Zhao et al. For whole tumor segmentation, our score of 0.785 indicates its ability to comprehensively delineate the tumor, which is crucial for guiding clinical interventions, although it does not achieve the highest score. In addition, the accuracy of our model for tumor core segmentation is demonstrated by a score of 0.778, indicating our model’s ability to distinguish the tumor core from surrounding tissue.

**Table 6:** Results comparison with other methods on the BraTS2019 validation dataset.

Method	Dice_ET	Dice_WT	Dice_TC
**Hamghalam et al.** (26)	0.725	0.897	0.795
**Minh et al.** (27)	0.784	0.903	0.811
**Kim et al.** (28)	0.672	0.876	0.764
**Zhao et al.** (29)	0.754	0.910	0.835
**Amian et al.** (30)	0.710	0.860	0.770
**Cheng et al.** (31)	0.777	0.902	0.824
**Islam et al.** (32)	0.704	0.898	0.792
**Proposed**	0.716	0.785	0.778

The BraTS2020 dataset, as summarized in **Table 7**, further contextualizes the performance of our model. With a score of 0.752, our model demonstrates improvements in the segmentation of enhancing tumor regions compared to Tarasiewicz et al. Although our model lags slightly behind the scores of Su et al. and Wang et al., it maintains a competitive stance. For whole tumor segmentation, our model achieves a score of 0.879, reflecting its robustness and validating its usefulness in clinical applications. Remarkably, our model’s dice score of 0.817 for tumor core segmentation outperforms several established models, reinforcing its accuracy and potential impact on treatment decisions.

**Table 7:** Results comparison with other methods on the BraTS2020 validation dataset.

Method	Dice_ET	Dice_WT	Dice_TC
**Tarasiewicz et al.** (33)	0.703	0.888	0.749
**Soltaninejad et al.** (34)	0.660	0.870	0.800
**David et al.** (35)	0.741	0.899	0.809
**Mchugh et al.** (36)	0.712	0.881	0.789
**Su et al.** (37)	0.754	0.896	0.821
**Wang et al.** (38)	0.787	0.901	0.814
**Zhao et al.** (39)	0.671	0.862	0.623
**Proposed**	0.752	0.879	0.817

Continuing this trend, the performance of our model on the BraTS2021 dataset is summarized in **Table 8**. Here, the proposed model achieves a dice score of 0.759, significantly outperforming the BraTS_Sg21 team and closely matching the @dgoon team. This underscores the refined ability of our model to accurately delineate enhancing tumor regions. For whole tumor segmentation, the model scores 0.803, placing it just below the top models and reinforcing its consistent performance. The score of 0.819 is particularly noteworthy, as it not only outperforms many established methods, but also underscores our model’s precision in isolating the tumor core, a critical region for prognostic and therapeutic considerations.

**Table 8:** Results comparison with other methods on the BraTS2021 validation dataset.

Team	Dice_ET	Dice_WT	Dice_TC
**BraTS_Sg21**	0.713	0.894	0.483
**DeepNet**	0.432	0.385	0.483
**@dgoon**	0.79	0.883	0.81
**@TecnmGTO**	0.554	0.739	0.597
**NiftyTorch_BraTS_21 **	0.078	0.705	0.533
**ColdBears**	0.759	0.895	0.836
**NefuBrain**	0.762	0.891	0.827
**Proposed**	0.759	0.803	0.819

In each iteration of the BraTS challenge, our model has demonstrated an impressive balance across all metrics, highlighting not only its consistency but also its evolution in performance. The network’s continuous learning framework ensures that our model adapts and potentially refines its segmentation capabilities with each dataset, which is critical for real-world clinical use. This sustained high level of performance across diverse and complex datasets illustrates the potential of the proposed network to serve as a reliable and efficient tool in the nuanced field of medical image segmentation.

The results of our study have significant implications for clinical practice in brain tumor management. The improved segmentation accuracy of our model promises to improve surgical planning and treatment strategies by providing more precise tumor delineation. This accuracy is critical for tailoring treatments to individual patients and could lead to better surgical outcomes by enabling more effective removal of tumors while sparing healthy tissue. In addition, the model’s high sensitivity in detecting small tumor regions can aid in early diagnosis, potentially improving treatment success rates and patient survival. The pseudo-labeling strategy using unannotated data provides a solution to the limited availability of annotated medical images, making the model particularly valuable in resource-constrained clinical settings. Finally, the robust performance of our model on diverse datasets suggests its applicability to different MRI scanners and protocols, underscoring its potential for widespread clinical adoption. Our research thus paves the way for more precise, personalized medical care in oncology, with the possibility of integrating this technology into existing medical imaging systems to improve patient outcomes.

Despite the advances our study offers in brain tumor segmentation using MRI images, it’s important to recognize its limitations. One notable limitation is the potential for dataset bias. The training and evaluation of our model, which was performed on datasets including BraTS2019, BraTS2020, BraTS2021, and a private dataset, may not fully capture the diversity of the global population, potentially affecting its generalizability. In addition, methodological biases inherent in our multiscale spatial distillation and pseudo-labeling strategies could affect the model’s performance, particularly when dealing with less common or atypical tumor types. This raises concerns about the adaptability of the model to a wide range of clinical scenarios. Furthermore, while our model addresses catastrophic forgetting, the delicate balance between retaining prior knowledge and adapting to new data may not always be optimal, which could affect overall performance. In addition, the computational requirements of our deep learning framework may pose a challenge in resource-constrained clinical settings.

## 7 Conclusion

In this study, we present a significant advance in brain tumor segmentation using multimodal MRI data through a novel continuous learning framework that combines multi-scale spatial distillation and pseudo-labeling strategies. Our approach addresses critical challenges such as unbalanced data categories and catastrophic forgetting, which are common in traditional segmentation models. The integration of the ResNet18 and DeepLabV3+ architectures not only improve feature extraction, but also optimizes model size, leading to increased accuracy and efficiency in tumor segmentation. These technical achievements have significant implications for improving the accuracy of brain tumor diagnosis and treatment planning, thereby impacting patient care. Our study of brain tumor segmentation from MRI images paves the way for several focused research directions. Future efforts should focus on increasing the diversity of datasets to cover a wider range of demographics and tumor types, thereby improving the generalizability of segmentation models. Exploration of innovative deep learning methods and optimization of continuous learning models are critical to address the dynamic nature of tumor characteristics. Integrating these models with clinical decision support tools can significantly impact diagnosis and treatment planning. In addition, developing computationally efficient models is essential, especially for resource-limited settings, while ensuring ethical and regulatory compliance in the application of AI in healthcare. Together, these areas provide a pathway for advancing the field of medical imaging and patient care.

## Data availability statement

The datasets presented in this article are not readily available because the raw and processed data necessary to replicate these study results are part of an ongoing research project and therefore cannot be shared at this time. Requests to access the datasets should be directed to RL, drlrp2022@163.com.

## Ethics statement

The research protocols for this study were carefully reviewed and subsequently approved by the Institutional Review Board (IRB) of both the Hangzhou Third People’s Hospital and the First Affiliated Hospital of Zhengzhou University. In accordance with ethical guidelines, written informed consent was obtained from all participants prior to their participation in the study. The research team affirms that all methods were conducted in strict accordance with the relevant guidelines and regulations.

## Author contributions

This study was conceived and organized by RL, JY, YH, WJ, PX, and LG. The literature search was conducted by RL, JY, and WJ. Data curation and interpretation were performed by RL, YH, PX, and LG. RL, YH, and WJ were responsible for the visual presentation of the data. Software execution was performed by RL, PX, and LG, and preparation of tables and figures was performed by RL, JY, YH, WJ, and LG. The first draft of the manuscript was written by RL, PX, and LG and subsequently refined and edited by RL, JY, and YH. All authors contributed to the article and approved the submitted version.
